# Sleep and Food Choice in a Dutch Student Population

**DOI:** 10.5334/jcr.ag

**Published:** 2015-07-13

**Authors:** Cathalijn H.C. Leenaars, Inge P.M. Klinkenberg, Audrey Aussems, Nedim Borger, Vivian Faatz, Anneloes Hak, Ellen Houben, Joyce Ramackers, Daphne Snackers, Andries Kalsbeek

**Affiliations:** Dept. of Anaesthesiology, Faculty of Medical and Health Sciences, University of Auckland, Auckland, New Zealand; Faculty of Psychology and Neuroscience, Maastricht University, Maastricht, the Netherlands; Dept. of Psychiatry and Neuropsychology / Alzheimer Center Limburg, School for Mental Health and Neuroscience, Faculty of Health, Medicine and Life Sciences, Maastricht University, Maastricht, the Netherlands; Dept. of Hypothalamic Integration Mechanisms, Netherlands Institute for Neuroscience (NIN), an institute of the Royal Netherlands Academy of Arts and Sciences, Meibergdreef 47, 1105BA, Amsterdam, the Netherlands; Dept. of Endocrinology and Metabolism, Academic Medical Center (AMC), University of Amsterdam (UvA), Meibergdreef 9, 1105AZ, Amsterdam, the Netherlands

**Keywords:** sleep diary, food diary, fat, protein, carbohydrates

## Abstract

**Background:** The increased risk of obesity among short sleepers is most likely explained by increased energy intake. However, food intake could not only be altered quantitavely but also qualitatively. Therefore, we performed a correlational analysis on self-reported food intake and sleep in 51 students from Maastricht and surroundings.

**Results:** Students that slept longer had a lower caloric intake: ρ = −0.378, p = 0.006, the amount of calories consumed per minute awake remaining relatively stable. However, sleep duration did not correlate with intake of percentage fat, saturated fat, carbohydrates or protein. Average energy intake during the reported breakfasts, lunches, dinners or snacks separately did also not correlate with total sleep time.

**Conclusion:** It seems that shorter sleep correlates with absolute caloric intake, but not with the intake of specific dietary components.

## Introduction

Less than one-half of people in the United States, Canada, Mexico, the United Kingdom, Germany and Japan are sleeping well every night, and 7–21 per cent of the population sleeps <6 h per night [1]. According to a meta-analysis of 30 studies, there is a consistent increased risk of obesity among short sleepers (≤5 h per night) [2]. The correlation between short sleep and increased obesity risk is also observed in children and adolescents [2,3,4,5,6,7,8]. Although energy metabolism may be affected by sleep curtailment, sleep restriction does not seem to have substantial effects on energy expenditure [9], while experimentally decreasing sleep (duration or quality) increases hunger and appetite [10]. Therefore, increased energy intake remains the most prevailing explanation for the association between short sleep duration and increased Body Mass Index (BMI)[11].

Besides quantity, quality of food and timing of food intake may be relevant when considering the relationship between sleep and body weight. Indeed, a link between sleep duration and food choices has been reported [12,13,14,15,16]. Sleep duration has been observed to correlate with snacking [12,9], irregular eating, excessive seasoning of food, insufficient consumption of vegetables [13] and consumption of energy-rich foods [14]. Besides, sleep deprivation alters the motivation underlying food choice; foods are chosen less for health and weight control [15]. Short-sleeping (<6h/day) female students had increased intake of calories and carbohydrates and decreased intake of fibers, fruit, whole grains and beans compared to those sleeping longer [16].

Because sleep may affect food intake qualitatively, research addressing food choice behaviours is paramount. While sleep deprivation in students is common, relatively few studies have adressed food choice in relation to sleep in students [16]. Besides, weight status, energy-balance related behaviours and sleep patterns differ between countries [17]. To investigate if sleep duration is associated with changes in food choice in a Dutch student population, we performed a correlational analysis.

## Materials and Methods

### Subjects & Procedure

The study was approved by the Ethics Comittee Psychology of Maastricht University. Written informed consent was obtained.

Initially, second-year psychology students aged 18–50 years were asked to participate in this study. As the target sample size was not easily reached, recruitment was extended to include other students. Second-year psychology students received course credits in exchange for their participation. Other students participated on a voluntary basis.

### Sleep- and Food Diaries

Participants kept a detailed food- and sleep diary for one week.

The sleep diary contained daily questions on the time of going to bed, time of waking up, number of nightly awakenings, total sleep duration and daytime napping, to be completed each morning after awakening, on paper or in word processing software by choice of the participant. For the present study, only the questions “How long did you sleep overall (hours)?” and “How long did you nap yesterday (hours)” were used to extract total sleep times. Total sleep times were averaged over the week to yield a robust estimate. While extracting data, internal consistency of the provided answers was verified. Ten per cent of the extracted data was checked for accuracy versus the original diaries by another experimenter.

As a food diary, we used the ‘eetmeter’ application on the website of ‘Het Voedingscentrum’ (the Dutch authority providing independent information on healthy, safe and durable food choices, https://mijn.voedingscentrum.nl/nl/eetmeter/). Participants were instructed to create an account and register all their intake over the course of the week that they kept their sleep diary. The “eetmeter” contains most food items available on the Dutch market, which can be entered separately in the chosen quantities for breakfast, lunch, dinner and snacks. Participants shared their password with the experimenters; passwords were kept until data had been extracted and verified.

After completion we extracted the following data on daily food consumption: amount of energy (kcal), fat (g), saturated fat (g), carbohydrates (g) and protein (g). Values for fat, saturated fat, carbohydrates and protein were converted to percentages of daily energy intake [e.g. 18]. Values were then averaged over the week to yield a robust estimate. Ten per cent of the extracted data was controlled for accuracy versus the original diaries by another experimenter.

### Data Analysis

Data were analysed using Excel (2007 & 2010) and SPSS (version 20 & 22). All data are presented as mean ± standard error of the mean (SEM).

Pearson’s correlation coefficient was calculated for average total sleep time and energy (kcal), fat (per cent including saturated fat), saturated fat (per cent), carbohydrates (per cent) and protein (per cent). The α-value indicating statistical significance was Bonferroni-corrected to 0.01 for these 5 primary analyses to decrease the possibility of a type-I error.

The target sample size (n = 50) was based on an *a priori* power analysis performed in G-Power [19] with a power of β = 0.8 for a two-tailed correlational analysis with an expected correlation coefficient of 0.45 and α = 0.01.

A number of sensitivity analysis were performed on the diary data: Pearson’s correlation coefficient was calculated for: a) average sleep time during the night, (excluding daytime naps), and energy (kcal), fat (per cent), saturated fat (per cent), carbohydrates (per cent) and protein (per cent); b) average total sleep time and energy intake (kcal) divided by time awake; and c) total sleep time and energy consumed (kcal) during breakfast, lunch, dinner and snacks.

## Results

### Demographics

Fifty-one students from Maastricht and surroundings participated in the correlational study, 45 of these (89 per cent) were second year psychology students. Average reported age was 22.3 years (n = 49, SEM = 0.60). Average reported height was 1.74m (n = 51, SEM = 1.18). Average reported BMI was 22.2 (n = 51, SEM = 0.46). Sex was registered for all participants; 71 per cent female, 29 per cent male.

### Sleep Diary Data

Sleep diaries were fairly complete; two students did not log 1 day each. Sleep duration on the subsequent night did not seem longer than usual for these 2 subjects. Averages were taken for the reported 6 days for these 2 students.

Average sleep duration was 7.9h ± 0.1 per night and 8.0h ± 0.1 per 24h (including daytime naps). Twenty-two students reported daytime napping; 14 had 1 nap during the week, 8 had multiple (6 × 2, 1 × 3, 1 × 5).

### Food Diary Data

All participants registered food intake for all seven days. Ten students did not report 17 breakfasts; 24 students did not report 53 lunches, and 8 students did not report 8 dinners. Underreporting can explain part of the missing meals, but students could also have skipped meals. Averages were taken for the reported meals; skipped or non-reported meals were ignored.

Average reported daily food consumption in the food diary was 1777 ± 67 kcal. Average caloric intake was 385 ± 20 kcal for breakfasts, 427 ± 21 for lunches and 727 ± 37 for dinners. Energy was mainly consumed as carbohydrates (45.1 per cent ± 0.9), followed by fat (32.4 per cent ± 0.7) and protein (17.3 per cent ± 0.6). Saturated fat comprised 11.9 per cent (± 0.4) of average daily energy intake.

### Correlation between Real-Life Food Intake and Sleep Duration from the Diaries

**Figure1:**
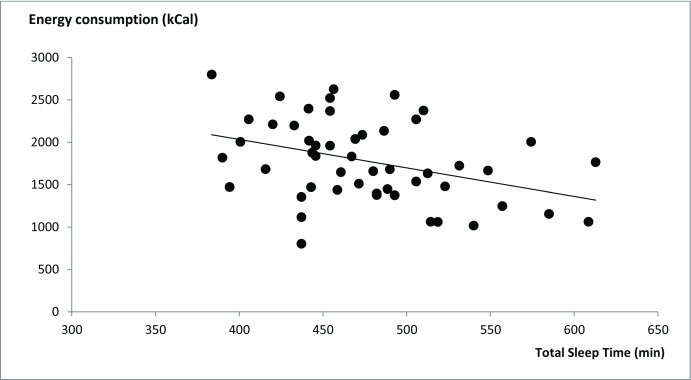
Negative correlation between average daily energy intake and average total sleep duration in a sample of 51 Dutch students (r = −0.378, p = 0.006). Energy intake is the individual daily average over 7 days as reported in a food diary. Total sleep time is the individual daily average sleep time including daytime naps as reported in a sleep diary.

The main correlational analyses showed a significant negative correlation between energy intake and total sleep duration: r = −0.378, p = 0.006 (Figure 1). On average, students that slept more had a lower caloric intake. None of the other correlations were significant (p > 0.02, Table 1).

**Table 1:** Correlational analyses on the food and sleep diary data.

Analysis	r	p

Primary analyses: total sleep time		
total sleep time (including daytime napping) & total energy intake (kcal)	−0.378	0.006*
total sleep time (including daytime napping) & fat intake (%)	0.132	0.355
total sleep time (including daytime napping) & saturated fat intake (%)	0.320	0.022
total sleep time (including daytime napping) & carbohydrate intake (%)	0.090	0.530
total sleep time (including daytime napping) & protein intake (%)	−0.283	0.044
Sensitivity analyses: nighttime sleep		
nighttime sleep & total energy intake (kcal)	−0.379	0.006
nighttime sleep & fat intake (%)	0.148	0.301
nighttime sleep & saturated fat intake (%)	0.270	0.055
nighttime sleep & carbohydrate intake (%)	0.069	0.631
nighttime sleep & protein intake (%)	−0.261	0.064
Sensitivity analysis: total sleep time and energy intake (kcal) divided by time awake		
total sleep time (including daytime napping )& energy intake (kcal) per awake minute	−0.187	0.190
Sensitivity analyses: total sleep time and energy consumed during different meals		
total sleep time (including daytime napping) & energy intake (kcal) during breakfast	−0.228	0.104
total sleep time (including daytime napping) & energy intake (kcal) during lunch	−0.215	0.125
total sleep time (including daytime napping) & energy intake (kcal) during dinner	−0.206	0.142
total sleep time (including daytime napping) & energy intake (kcal) as snacks	−0.214	0.128

Sensitivity analyses restricted to night-time sleep duration (excluding daytime naps) also showed a negative correlation between energy intake and sleep duration (r = −0.379, p = 0.006). None of the other correlations were significant (p > 0.05, Table 1).

Shorter sleep durations could bring about higher energy expenditure because of longer activity. Therefore, we divided the average daily energy intake by average daily time awake. These values did not correlate with total sleep time (r = −0.187; p = 0.190).

As increased energy consumption could be specifically consumed during certain meals, we repeated the main analysis for energy consumption during the different meals separately. Average energy intake during the reported breakfasts, lunches, dinners or snacks separately did not correlate with total sleep time (p > 0.10).

## Discussion

We found a negative correlation between average reported daily caloric intake and sleep duration in a small student sample, both for total sleep time and for time slept during the night. These results are in line with a number of preceding studies also showing a negative correlation between sleep duration and food intake [e.g. 12,13]. From our results it is not clear if short sleep results in more (time for) eating or if eating more results in sleeping less.

Only few experimental studies have shown an effect of diet on subsequent sleep. For example, the amino acid tryptophan seems to have a beneficial effect on subsequent sleep in infants and insomniacs [20,21]. Evidence from experimental weight loss studies with decreased sleep duration at baseline [22,23] remains inconclusive; in one study, sleep duration remained decreased after diet-induced weight loss (n = 6) [22], in the other study, the sleep distribution nearly normalised (n = 6 adolescents) [23].

Experimental sleep restriction did increase food intake and body weight gain in a large sample of healthy adults [24], and hunger and appetite in a sample of 12 healthy young men (average age 22 years) [25]. Longitudinal observational studies adressing body mass index and sleep duration show that a decreased sleep duration increases the odds for overweight and / or obesity at a later stage [3,26,27,28,29,30]. In our young adult sample it seems likely that shorter sleep duration precedes increased caloric intake.

A small number of previous studies showed a correlation between shorter sleep duration and increased snacking, irregular eating, excessive seasoning of food, insufficient consumption of vegetables and craving for high-calorie food [12,13,31]. We did not find a correlation between total sleep time and caloric intake for separate meals or snacks.

As shorter sleep durations go together with longer periods of activity, energy expenditure will theoretically increase, and caloric intake would need to increase to compensate for this higher total energy expenditure. In our healthy non-obese student population, the amount of energy intake per time awake did not correlate with the total sleep time, indicating a relatively stable energy intake per time awake. This observation could explain why we and others observe a clear correlation between food intake and sleep duration overall, while the risk of obesity in relation to sleep seems to be relatively small and limited to extremely short sleepers [32,33].

Previous studies adressing the relationship between food intake and sleep in young adults have not investigated the effect of *daytime* sleep, but studies in children and infants show limited effects of daytime sleep on overweight and obesity [34].

Limitations of this study are its small sample size, and the inclusion of non-obese participants only, both due to our preferential sampling of second-year psychology students. Future studies should confirm our findings in a larger sample. For future studies, it would be of interest to assess the timing of food intake, as this could also affect obesity risk [e.g. 35].

On average, students eating more were awake longer. Because we did not find any correlations between sleep duration and the percentages of energy intake as fat, carbohydrates or protein, it seems that shorter sleep is more strongly related to absolute caloric intake than to intake of specific dietary components.

## Competing Interests

The authors declare that they have no competing interests.
